# Supplementary material for: Acceptance and compliance with micronutrient powder (MNP) among children aged 6–23 months in northern Nigeria
Source: PLOS Glob Public Health. 2022 Oct 17;2(10):e0000961. doi: 10.1371/journal.pgph.0000961 (PMC10022258; doi:10.1371/journal.pgph.0000961)
Supplement: S1 Dataset — (ZIP) [file pgph.0000961.s008.zip › S8_Dataset/Direct Observations.zip!/Direct Observations/060715_K_J_AD_2_DO.pdf]

1.1.1 Instrument F –Direct Observation Form

<<Instrument F Direct Observation Form>>
**PI Name:** PragyaMathema,Stephen Kodish<br>**Study Title:** Formative research to develop an infant and young child feeding (IYCF) intervention with micronutrient powder (MNP) for children aged 6 – 23 months<br>**PI Version/Date:** Version1.0/January 24, 2015

Date:06/07/15 Location: Rugga

Data Collector: Amina Duniya

Main goals of this direct observation are to answer the following questions:<br><br>1. In <u>this</u> household, what would be the best way(s) to give nutritional supplements to <u>young children</u>? Why?<br><br>2. In <u>this</u> household, describe the food sharing that you observe throughout the meal. Are there any <u>predictable</u> patterns?<br><br>3. In <u>this</u> household, who is favored and how is this favoritism expressed during meal preparation and eating?

Household Composition Table (Fill out <u>as</u> you do data collection)

INITIALS	SEX (M/F)	AGE (estimated)	Relationship to household	Description (not necessary)
BM	F	18years	Mother of the baby	Slim, dark in complexion, wearing beads on her neck and hands
BB	M	12months	Baby of the house	Yellow top with no trouser, yellow in complexion
GM	F	50years	Grandmother	Slim, light in complexion, wearing a green blouse and wrapper
SS	M	3years	Senior son in the house	Light in complexion and wearing a blue kaftan top
BF	M	25years	Baby's father	Black kaftan, tall and light in complexion

TIME	ACTOR INITIALS	RECIPIENT INITIALS	WHAT HAPPENED	FOOD	Reactivity
9:00	BM	BB	BB sleeping on her back		
9:15	BM	BB	Gives him the MNP mixed with cold kunun gero (a sachet)	Kunun gero	
9:30	BM	BB	Breast feeding BB	Breast milk	
9:35	BB		Playing on BM's lap		
9:40	BM		Making beads		
9:50	BM		Still making beads		
	BB		Still playing		
10:00	BM	BB	Breastfeeding the baby	Breast milk	
10:05	BM	SS	Gives baby to SS to play with		
	BB		Refuses to stay with SS		
	BM		Collects him back from SS		
10:10	BB	BM	Playing with BM		
10:20	BB		Still playing		

TIME	ACTOR INITIALS	RECIPIENT INITIALS	WHAT HAPPENED	FOOD	Reactivity
10:27	BB		Crying for food		
10:30	BM	BB	Breastfeeding BB	Breast milk	
10:40	BB		Starts crying again		
	BM	BB	Breastfeeding baby	Breast milk	
10:50	BB		Still sucking	Breast milk	
10:55	BB		Sleeping		
11:00	BB		Still sleeping		
11:10	BB		Still sleeping		
11:15	BB		Still sleeping		
	BM		Making beads while BB sleeps		
11:20	BB		Still sleeping		
11:30	BB		Still sleeping		
11:40	BB		Wakes up from sleep		
	BB		Playing on the mat		

TIME	ACTOR INITIALS	RECIPIENT INITIALS	WHAT HAPPENED	FOOD	Reactivity
	BM		Still making beads		
11:45	BB		Starts to cry		
	BM	BB	Breastfeeds BB	Breast milk	
11:50	BB		Finished sucking and starts to play		
	BM	BB	Gives him kunu gero to drink	Kunun gero	
11:55	BM	BB	Still feeding the baby	Kunun gero	
	BB		Finished drinking kunu and starts to play on the mat	Kunun gero	
12:00	BB		Still playing		
12:10	BB		Still playing		
12:20	BB		Still playing		
12:30	BB		Still playing		
12:40	BB		Starts crying		
	BM	BB	Breast feeds him	Breast milk	
	BB		playing		
12:50	BB		Still playing		
	BM	BB	Feeds him with kunun gero	Kunun gero	

TIME	ACTOR INITIALS	RECIPIENT INITIALS	WHAT HAPPENED	FOOD	Reactivity
12:55	BB	SS	Playing with SS on the mat		
1:00	BB		Starts to cry		
	BM		Beast feeds BB		
	BB		Refuses to suck and begins to play		
1:10	BB		Still playing		
	BM		Sleeping		
1:15	BB		Still playing		
1:20	BB		Still playing		
	BM		Still sleeping		
1:30	BB		Still playing		
	BM		Still sleeping		
	BB		Starts crying		
	BM	BB	Beast feeds BB	Breast milk	
1:35	BB		Sucking and sleeping	Breast milk	
1:40	BB		sleeping		
	BM		sleeping		
1:50	BB		Still sleeping		
2:00	BB		Still sleeping		
	BM		Still sleeping		

TIME	ACTOR INITIALS	RECIPIENT INITIALS	WHAT HAPPENED	FOOD	Reactivity
2:10	BB		Still sleeping		
2:20	BB		Still sleeping		
	BM		Still sleeping		
2:30	BB		Still sleeping		
2:40	BB		Still sleeping		
	BM		Still sleeping		
2:48	BB	BM	Wakes up and starts to wake the mother		
	BM	BB	Carries BB and puts him on he back		
2:55	BM		Carries guinea corn from outside the house to the bedroom. BB is still strapped to her back		
3:05	BM		Still fetching guinea corn with the baby strapped to her back		
3:10	BM		Still fetching guinea corn with the baby strapped to her back		
3:20	BM		Still fetching guinea corn with the baby strapped to her back		
3:25	BM		Pounding guinea corn		
3:35	BM		Still fetching guinea corn with the baby strapped to her back		
3:45	BM		Still fetching guinea corn with the baby strapped to her back		
3:50	BM		Still fetching guinea corn with the baby strapped to her back		

TIME	ACTOR INITIALS	RECIPIENT INITIALS	WHAT HAPPENED	FOOD	Reactivity
3:55	BM	BB	Breast feeds baby	Breast milk	
4:00	BM		Sieves pounded guinea corn in other to remove the back with BB strapped to her back		
4:10	BM	BF	Hands BB to him to hold		
	BB		Refuses to stay with BF		
	BM	BB	Collects him and breast feeds baby	Breast milk	
4:15	BM	BB	Finished feeding him and straps him to her back		
4:20	BM		Still pounding guinea corn		
4:30	BM		Continued pounding guinea corn with baby strapped to her back		
4:40	BM		Still pounding guinea corn		
	BB		Starts crying on her back		
4:45	BM	BB	Brings him down and breast feeds him	Breast milk	
4:50	BM	BB	Finished breast feeding him and puts him down and continues pounding		
4:55	BB		Still playing on the floor		
	BM		Still pounding		
5:00	BB		Still playing		
	BM		Still pounding		

Primary Questions (Complete after you complete data collection):

From what you have seen observing THIS HOUSEHOLD, please answer the following questions in detail:

Question	Write your detailed response here
1. In this household, what would be the best way(s) to give nutritional supplements such as an MNP to young children to ensure compliance?<br><br>Why?	The best way to give this product to ensure compliance is through the grandfather. He usually has the final say in the house. The grandmother too is very important because the mother of the child acts according to what she asks her to do and the grandmother takes order from the grand father
2. In this household, who is favored during meal preparation or feeding?<br><br>How is this favoritism expressed?	The baby because he is always with the mother and she feed her directly.
3. How could we promote the MNP supplement for this family that would attract their attention?<br><br>Who would they listen to?	Through the grandfather who is is the practical head of the entire clan. Whatever he says holds

4. In this household, what would be the best ways to promote the MNP but limit how much it would be shared?<br><br>Why?	By educating the grandfather on the importance of the product to the child and what he stands to lose if it is shared with others<br><br>I believe the grandfather is the person they all listen to
5. What other issues and concerns did you see with this family that are important for us to consider with regard to introduction of an MNP?<br><br>Why?	The mother feeds the baby with breast milk most of the time. The only supplementary feeding she does for him is with the kunun gero which is not often
6. Based on your experience observing this child today, do you believe reactivity negatively impacted your ability to see the food-related behaviors of the child?<br><br>Please explain your answer in detail.	No, there was no reactivity that I noticed at all. The only thing I noticed was that the mother of the child does not take decisions on her own; it is the mother in-law (grandmother) that speaks for her.
